# Unique knowledge, unique skills, unique role: Aboriginal and Torres Strait Islander Health Workers in Queensland, Australia

**DOI:** 10.1136/bmjgh-2021-006028

**Published:** 2021-07-02

**Authors:** Stephanie M Topp, Josslyn Tully, Rachel Cummins, Veronica Graham, Aryati Yashadhana, Lana Elliott, Sean Taylor

**Affiliations:** 1 College of Public Health, Medical and Veterinary Sciences, James Cook University, Townsville, Queensland, Australia; 2 Nossal Institute for Global Health, University of Melbourne, Melbourne, Victoria, Australia; 3 Torres and Cape Hospital and Health Service, Cairns, Queensland, Australia; 4 Centre for Health Equity Training Research & Evaluation, University of New South Wales, Sydney, New South Wales, Australia; 5 Ingham Institute for Applied Medical Research, Sydney, New South Wales, Australia; 6 School of Public Health and Social Work, Queensland University of Technology, Brisbane, Queensland, Australia; 7 Top End Health Service, Northern Territory Department of Health, Casuarina, Northern Territory, Australia; 8 Menzies School of Health Research, Darwin, Northern Territory, Australia

**Keywords:** health systems, health services research

## Abstract

Aboriginal and Torres Strait Islander Health Workers (A&TSIHWs) are a professional cadre of Australian health workers typically located in primary care clinics. The role is one of only two that is ‘identified’— that is, it must be occupied by an Aboriginal and/or Torres Strait Islander person — and holds specific responsibilities in relation to advocating for facility-level cultural safety. However, lack of understanding of the distinctive skills, scope and value associated with the A&TSIHW role remains pervasive in the broader health workforce. Positioned to represent the perspective of those working as A&TSIHWs, and drawing on 83 in-depth interviews with A&TSIHWs and others, this qualitative study reports on the core functions and distinctive orientation of the role, and seeks to articulate its distinctive value in the modern Queensland health service. Findings highlight the multifaceted (generalist) nature of the A&TSIHW role, which comprises three core functions: health promotion, clinical service and cultural brokerage. Underpinning these cross-cutting functions, is the role’s unique orientation, defined by client-centredness and realised through Indigenous strengths based ways of knowing, being and doing. The findings highlight how the A&TSIHW role is one of the only mechanisms through which Aboriginal and Torres Strait Islander knowledge can be brought to bear on context-specific adaptations to routine health service practices; and through which the impacts of lack of cultural or self-awareness among some non-Indigenous health professionals can be mitigated. The complexity of such work in a government health system where a dominant biomedical culture defines what is valued and therefore resourced, is under-recognised and undervalued and contributes to pressures and stress that are potentially threatening the role's long-term viability.

**Key questions**

**What is already known?**

- In the state of Queensland the role of *Aboriginal and/or Torres Strait Islander Health Worker* (A&TSIHW) is one of only two positions that must be occupied by an Aboriginal and/or Torres Strait Islander person.
- Typically located in primary care clinics, A&TSIHWs are members of integrated teams of health professionals responsible for helping build client engagement and self-management within a framework of culturally safe care.
- A&TSIHWs are one of only two roles with explicit responsibility for advocating cultural safety at the whole-of-service level, not simply as part of individual practice.

**What are the new findings?**

- The A&TSIHW role is multifaceted and comprises three core functions: health promotion, clinical service and cultural brokerage.
- Cross-cutting functions help A&TSIHW place the client/person—rather than disease—at the centre of their work.
- Both the cross-cutting functions and distinctive client-centred orientation of the A&TSIHW role are realised through Indigenous strengths based ways of knowing, being and doing.

**What do the new findings imply?**

- A&TSIHWs are unique in their the ability to, and formal responsibility for, holding both biomedical and Aboriginal and Torres Strait Islander knowledges and applying them as appropriate to shape service delivery to Aboriginal and Torres Strait Islander clients.
- The A&TSIHW role is one of the only mechanisms available in frontline services through which active adaptations can be made to otherwise biomedically driven practices in order to account for the different knowledge systems that shape Aboriginal and Torres Strait Islander peoples' understanding of health.
- These service adaptations also represent one of the only ways to effectively confront and mitigate the effects of continuing lack of cultural or self-awareness among some non-Indigenous health professionals.

## Introduction

In Australia, provision of healthcare to Aboriginal and Torres Strait Islander peoples, *by* Aboriginal and Torres Strait Islander health providers is known to improve quality of care and health outcomes.^1–3^ To that end, most states and territories in Australia articulate a policy goal of greater participation by Aboriginal and Torres Strait Islander peoples in the health workforce including in a range of professional roles such as nurses, midwives, allied health professionals and doctors. In the context of Aboriginal and Torres Strait Islander health, however, the role of *Aboriginal and/or Torres Strait Islander Health Worker* (A&TSIHW) takes on particular significance, being one of only two ‘identified’ roles. An identified role is one that can only be occupied by an Aboriginal and/or Torres Strait Islander person, and typically brings with it specific (rather than incidental) responsibilities for ensuring the delivery of culturally safe services. The defining objective of this study was to examine—from the perspective of those in the role—the distinctive functions and orientation of A&TSIHW, and their unique value in the modern Australian health system. In the remainder of this paper and to help aid recognition of the position of A&TSIHW, we respectfully use the acronym A&TSIHW.

Typically, although not exclusively located within primary care clinics, A&TSIHWs are meant to be members of integrated teams of health professionals responsible for helping build client engagement and self-management within a broader framework of holistic and culturally safe care.^1 4^ The Australian Health Practitioner Regulation Agency defines culturally safe practice as ‘the ongoing critical reflection of health practitioner knowledge, skills, attitudes, practising behaviours and power differentials in delivering safe, accessible and responsive healthcare free of racism’.^5^ Partly reflective of this intent, state health departments such as Queensland Health define the A&TSIHW role (see box 1) as an ‘advocate for the delivery of services in accordance with the Cultural Respect Framework for Aboriginal and Torres Strait Islander Health 2004–2009 (Australian Health Ministers’ Advisory Council)’.^6^ Inclusion of this advocacy role vis-à-vis cultural safety is important. It means A&TSIHWs, alongside their hospital counterparts Indigenous Hospital Liaison Officers, are the only front-line professionals in the state health workforce whose job includes responsibility for advocating culturally safe care at the whole-of-service level, and not simply as part of their individual work practice.

Box 1 **Definition of Aboriginal and Torres Strait Islander Health Worker in the Queensland Health Aboriginal and Torres Strait Islander Health Worker Career Structure 2009^4^**

A Queensland Health Aboriginal and Torres Strait Islander Health Worker is an Aboriginal or Torres Strait Islander person who:

- Works within a primary healthcare framework to achieve better health outcomes and better access to health services for Aboriginal and Torres Strait Islander individuals, families and communities.
- Is required to hold the specific Aboriginal and Torres Strait Islander primary healthcare qualification.
- Advocates for the delivery of services in accordance with the *Cultural Respect Framework for Aboriginal and Torres Strait Islander Health 2004–2009 (Australian Health Ministers’ Advisory Council*).

Aboriginal and Torres Strait Islander Health Worker positions are ‘identified’ positions (see IRM 1.13–12). There is a genuine occupational requirement that the occupants of these positions are Aboriginal or Torres Strait Islander.

Notwithstanding the unique nature of the A&TSIHW role within Australia’s state-based health workforces, lack of understanding of the distinctive skills, scope and value associated with its function remains pervasive.^7–18^ This manifests in different ways including poor recognition of the role’s purpose and scope^7 14^; misunderstanding of role boundaries^19 20^; lack of respect for reporting lines; and in demands that A&TSIHWs assist other professionals with often menial tasks.^10^ These factors coalesce in the lack of any clear models of care that operationalise the ‘ideal’ of supporting the profession as a member of a multidisciplinary team. Empirical papers and commentaries spanning several decades and different jurisdictions, reviewed in 2018,^21^ demonstrate the A&TSIHWs’ role has devolved in some cases to ‘glorified taxi drivers’.^9 13 14^ The same review observed a common assumption that the A&TSIHWs’ primary function was to facilitate non-Indigenous health professionals' own professional practice, rather than engage in distinctive work that could improve the acceptability, accessibility and appropriateness of services for Aboriginal and Torres Strait Islander clients.^3 16^ Such pervasive lack of understanding of the A&TSIHW role has been described as leading to lower self-worth and high levels of stress among those working as A&TSIHWs, damaging individual career trajectories, role retention and ultimately service-wide cultural safety.

This study emerged as part of a larger project designed to answer the question: ‘*what barriers exist to the full participation of A&TSIHW in the state (Queensland) health system?*’ The study arose from a collaboration between the first, second and senior authors—representing academic, A&TSIHW management and practitioner perspectives, respectively—following discussions about the long-term viability of the A&TSIHW role in the context of multiple and systemic pressures. Recognising the marginalisation of the voices of those employed as A&TSIHWs, the project was designed to place A&TSIHW voices at the centre of enquiry. As one component of a broader suite of strategies to address that marginalisation, the objective of this work was to map—from the perspective of those working as A&TSIHWs—the unique purpose, functions and skills required, of A&TSIHWs.

Throughout this paper we respectfully use the terms Aboriginal and/or Torres Strait Islander to identify communities, peoples and individuals. We use ‘Indigenous’ where it forms part of a formal role or framework name (eg, Indigenous Hospital Liaison Officer) or if used in direct quotation, or to refer to non-Indigenous people. We use the acronym A&TSIHW to refer to the *role* held by many of the Aboriginal and/or Torres Strait Islander participants in this study.

## Methods

### Study setting

This study was undertaken in a Hospital and Health Service (HHS) in the state of Queensland. Queensland has the highest number of A&TSIHWs of any state or territory in Australia.^22^ At the time of writing, Torres and Cape HHS had 234 A&TSIHW positions of which 196 were occupied, comprising 131 (67%) permanent, 35 (17%) temporary and 30 (15%) casual employees.

### Study design

This study was codesigned and undertaken by a team which included Aboriginal (JT, RC), Torres Strait Islander (ST) and non-Indigenous (SMT, VG, LE, AY) collaborators. This study was undertaken with individuals working as A&TSIHWs, nurses and doctors and community members from towns linked to seven primary health centres and one hospital; as well as key informants and administrators with extensive knowledge of health services in the region. While time and resource constraints precluded using a participatory action approach, which would have been ideal, the study was designed to privilege the voices, experiences and lives of the A&TSIHW and their communities, placing their interests, experiences and knowledge at the centre of the enquiry. For this reason, qualitative methods were used and the study was conducted in four phases: (1) consultation with A&TSIHWs regarding overall study design and construction of an interview guide, (2) interviews and document review, (3) preliminary analysis and sharing and member checking of interpretation with all A&TSIHW study participants and (4) finalisation and reporting back of findings to all A&TSIHWs in the region, and other HHS stakeholders.

### Sampling

A total of 83 interviews were conducted with study participants comprising four groups: (1) current or former state-employed A&TSIHWs from the nominated study sites (n=51); (2) currently employed (non-Indigenous) clinical professionals working at one of the nominated study sites (n=19); (3) community members aged 18 years or more and resident in one of the communities linked to the study clinics (n=8) and (4) key stakeholders (n=5), comprising individuals with specific knowledge of health services and/or the A&TSIHW role in the state health system. Convenience sampling based on initial email communication, and subsequent consultation visits was used to recruit A&TSIHWs and non-Indigenous clinical professionals working in the study clinic sites. Participants were informed of the interview visit dates in advance and could choose to participate in an interview or not; for any individual who missed out on an in-person interview but wished to participate a phone interview was offered. Recruitment of community members was reliant on direct referral by local A&TSIHW, and snowball sampling where participants recommended and were willing to provide a direct introduction. Community interviews were only conducted where a direct introduction by an existing study participant was possible. Recruitment of key informants was purposive based on expertise or experiences, with invitations and subsequent interviews conducted either in person or over the phone. RC, JT and ST provided cultural advice and guidance throughout, but JT and ST were not directly involved in recruitment or interviews.

Participants took part in either an interview or a focus group. A&TSIHW interviews explored individuals’ motivations for working as an A&TSIHW; their understanding of the value and purpose of the role; and their experiences in the role in relation to regulatory, organisational and sociocultural pressures and expectations. Clinician interviews explored their understanding of the value and purpose of A&TSIHW, and perceptions regarding barriers and enablers to effective integration and performance in primary healthcare teams. Community member interviews explored their understanding of the value and purpose of the A&TSIHW role and perceptions of its utility in the context of current community health needs. Key stakeholder interviews asked similar questions to those noted above, but were tailored to the expertise of the individual and often included an additional focus on the impact of historical and recent policy and organisational reforms on the A&TSIHW role.

All bar five interviews were conducted face to face, and interviews ranged from 20 and 100 min. In four cases, and at the request of the participants, group interviews (with two, two, six and two participants, respectively) were held instead of individual interviews. Interviews were recorded with permission in all but four instances, where extensive notes were taken instead. Interviews were conducted by one Aboriginal (RC) and two non-Indigenous team members (SMT, VG) all experienced qualitative researchers. All interviews were conducted in English. Audiorecordings were transcribed verbatim and uploaded into NVivo V.12 (QSR International, 2015). Participants were provided with a verbatim copy of their interview transcript to check prior to analysis. Coding was inductive, with themes identified iteratively over several rounds and later examined for fit against Indigenous and other (eg, institutional governance) frameworks. Coded data were summarised and presented to A&TSIHWs in person or via video conference during which professional, contextual and cultural insights and critiques were provided and interpretations refined. In the results, to protect participant confidentiality, identification is limited to the participants’ location in either the Cape York, or Torres Strait and Northern Peninsula Area (NPA) regions and a sequentially assigned numerical ID.

### Patient and public involvement and analysis

Currently employed A&TSIHWs in participating clinics and community members were consulted during prestudy visits to each site. Visits tested the appropriateness of the concept and subsequently sought guidance on study design and data collection approaches including formulation of recruitment and dissemination. The project was a collaboration between researchers from James Cook University and the Aboriginal and Torres Strait Islander Health directorate of the Torres and Cape HHS. Aboriginal and Torres Strait Islander collaborators from the HHS were not involved in data collection, but provided intellectual and cultural leadership in study design, as well as advising on history, local context and culturally safe research strategies.

We took an iterative approach to analysis built around collaboration with A&TSIHWs at three critical stages including (1) during initial prestudy consultation and permissions sought from local councils or community groups; (2) in review of interview transcripts and provision of corrections or additions where study participants so chose and (3) in feedback and critique of the preliminary findings. Following these processes, initial rounds of analysis was grounded and inductive with coding led by SMT. Subsequent rounds of analysis considered preliminary nodes and themes against the conceptual framework of Indigenous Ways of Doing, Being and Knowing described in Askew *et al*^23^ who themselves cite Martin and Mirraboopa^24^ and were found to be a good fit. These concepts were subsequently used to organise and report findings overall.

No community members were directly involved in the study design. However both preliminary analyses and recommendations arising from the study findings were subject to consideration and critique by individuals in the A&TSIHW role following oral and written presentations, and final recommendations were developed with explicit consideration for the role of strength-based strategies and Indigenous Ways of Being, Ways of Doing and Ways of Knowing^23 24^ identified over the course of the study.

## Results

The state government role description for an A&TSIHW (see box 1) defines the job along three very broad parameters; (1) attainment of the required qualification (Certificate IV in Aboriginal and Torres Strait Islander Primary Healthcare); (2) the need to work within the primary healthcare framework to achieve better health outcomes and (3) responsibility for advocating for delivery of services in accordance with the Cultural Respect Framework. According to the majority of participants in this study, the definition falls short of explaining *what* it is that A&TSIHWs do and why it is distinctive. In the following, we aim to better characterise the key functions and orientation of the role in order to bring into sharper focus its distinctive nature.

Out of respect for the distinct nature of the regions we attribute quotes to individuals in the broad regions of Torres Strait and NPA, or Cape York Peninsula, rather than specific communities or clinics.

### Role function

**Figure 1:**
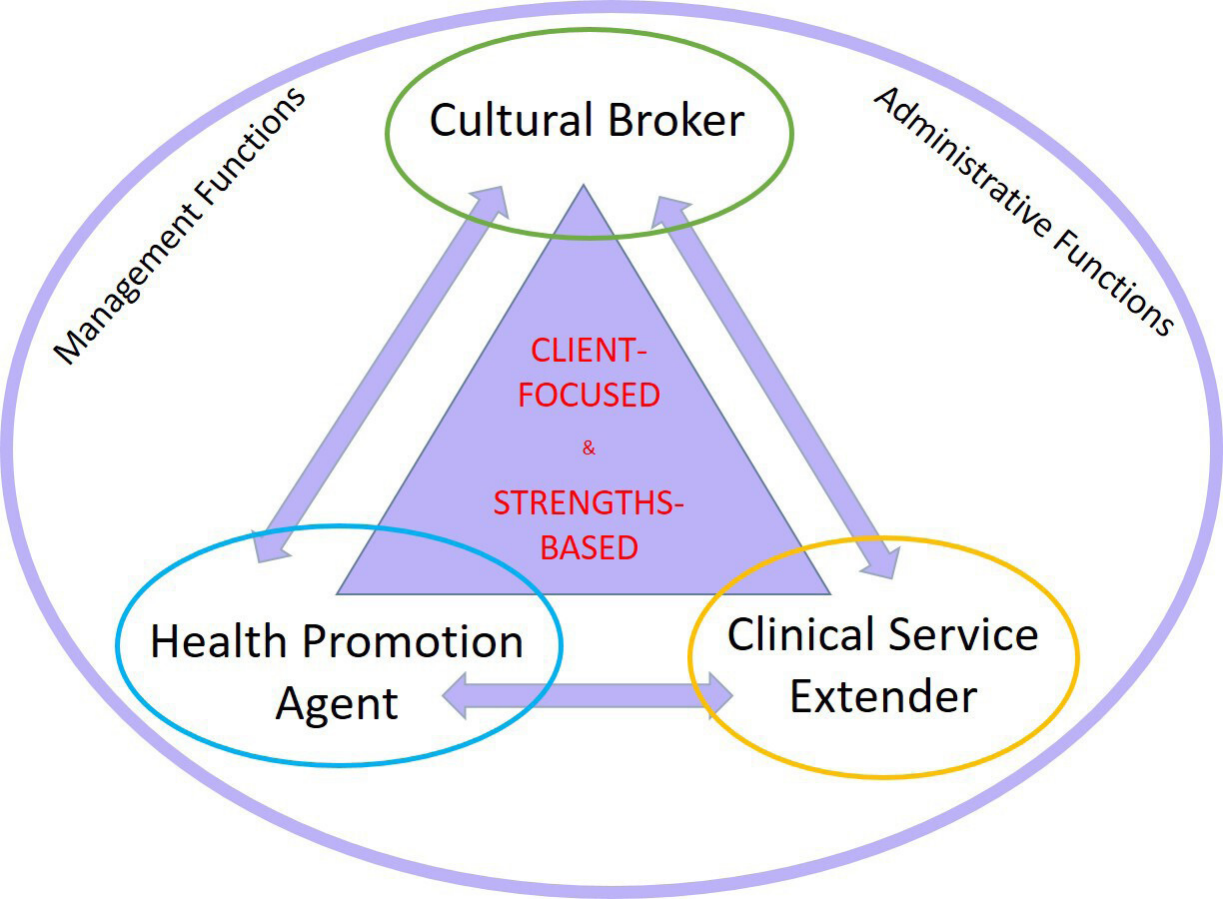
The tripartite functions of Aboriginal and/or Torres Strait Islander Health Workers.

A critical feature of the A&TSIHW role, as captured in every A&TSIHW interview in this study, was its multifaceted nature, which integrates biomedical, promotive and cultural knowledge and strategies to tailor communication and service activities for Aboriginal and Torres Strait Islander clients. Synthesising open-ended job descriptions from 51 A&TSIHWs, and cross-referencing those with 19 non-Indigenous health professionals, figure 1 captures the three core functions of A&TSIHWs, being cultural broker, health promotion agent and clinical service extender. Common administrative and managerial functions surround these core functions as they are not specific to the role, but nonetheless occupy substantial amounts of time for some in the A&TSIHW role.

While all A&TSIHW participants described engaging in elements of the three core functions in some way, the relative emphasis on each function differed from individual to individual. A focus on clinical management, health promotion and well-being, cultural brokerage, or in some instances management or administration, was influenced by four factors: qualification, location, manager and personal preference. With regards to qualification, although not recognised in the formal state government career structure, A&TSIHWs described pursuing qualifications over and above the basic requirement for a Certificate IV in Aboriginal and/or Torres Strait Islander Health Primary Healthcare in clinical specialties including in sexual health and mental health. Generally speaking, A&TSIHW with specialist qualifications and operating within programmes that matched their specialty described spending more time on clinical management.

Combined with qualification, however, the geographic location of the clinic and the degree of remoteness specifically was another important determinant. In the most remote facilities such as single-nurse posts of the outer islands of the Torres Strait region, A&TSIHWs were often the only permanent members of staff, resulting in significant burden of management, administrative tasks, in addition to emergency response, clinical service delivery, promotive activities and cultural brokerage. In more geographically central facilities with larger cadres of nurses and doctors, A&TSIHWs tended to have a fewer management responsibilities, and a narrower scope of clinical work, with greater emphasis on public health and health promotion activities arising from working in ‘programmes’ such as Chronic Disease, Sexual Health or Social and Emotional Well-being. Table 1 provides an illustrative and non-exhaustive list of clinical, health promotion and administrative functions as described by participants in this study.

**Table 1:** Illustrative areas of work (excluding cultural brokerage) for A&TSIHW participants

Clinical service extender	Sexual health
	Emotional/social well-being
	Maternal health
	First responder
	Palliative care
	Mental health
	Exercise planning
	Nutrition and meal planning
Public health and health promotion	Antenatal care
	Immunisation
	Ear health
	Eye health
	Scabies
	Rheumatic heart disease
Clinic management and administration	Clinic planning and management
	Reception duties
	Client transport requests
	Coordination of visiting clinical teams
	Transport

A third factor influencing A&TSIHWs’ focus and function was the experience and attitude of the facility manager towards this cadre. While some facilities in the most remote areas saw A&TSIHWs operating as facility managers themselves, management positions were more often held by nurses. A&TSIHW participants noted that the degree of autonomy and ability to tailor their work across the three core functions were heavily dependent on whether the facility manager understood their capabilities and supported their involvement and leadership. Many interviewees described the requirement that they take on administrative functions, a workload that was exacerbated by a lack of administrative positions in some services. These administrative functions could include the onerous task of coordinating travel with a central booking service, scheduling appointments and answering the phone at the clinic.

So here we don’t utilise the skills. We have got very educated [Aboriginal and Torres Strait Islander] Health Workers who probably don’t use their skills and then they end up migrating to a bit of admin and migrating to a bit of liaison so it is hard to get back. (Cape York, Health Worker, #11).

Finally, as may be expected in any profession, individual preferences and capabilities combined with specific job requirements all influence individual’s focus.

Notwithstanding the fluidity of role function described above, the importance of cultural brokerage was a common theme in the accounts of all A&TSIHW participants. Here we use the concept of cultural brokerage to capture both tangible activities and less tangible approaches to work, with the latter being explored further in the next section. Tangible activities that formed part of day-to-day work included translation, explanation and representation and, while not always health-specific, were designed to enable and enhance clients’ and community members’ access, understanding and benefit from health services. Table 2 synthesises these activities and provides illustrative quotes.

**Table 2:** Illustrative examples of cultural brokerage activities

Function	Types of activities	Illustrative quotes
Cultural brokerage	Interpretation (two-way)	*It’s very vital, especially for (A&TSIHW) to be present in an emergency situation in the A&E room because they can translate in language, and to the nurses and if there’s a doctor on here, and even just liaise for the community. (Cape York, Health Worker, #9*) *Yeah, translate sometimes, especially with [remote community] people that come in because they only speak their language out there. (Torres and NPA, Health Worker, #37*) *I said to the nurse navigator, you would need to get a family member to come in. And she said, no we’ll get the interpreter or the person that does hand signs and everything - sign languages to come in. And I said, sorry but he won’t understand white man’s sign languages. He understands our [community] sign languages. (Torres and NPA, Health Worker, #41*)
	Explanation (two-way)	*Sometimes [clients] don’t understand what the medication are they have to take, and my role is to explain what the doctors have told them if they don’t understand, I explain the medication, how they should take it, when they should take it and why they should take it. Plus any other issues that they have, social or any emotional issues as well. (Cape York, Health Worker, #1*) *And liaising with visiting teams as well in the health worker role (…) giving community feedback and consulting with community if new services are coming in, explaining to them. (Cape York, Health Worker, #3*)
	Representation (two-way)	*Yep, I come in the morning, first thing in the morning, we have a team meeting handover, and as the Indigenous person, I just speak what I hear in the community. And my role is to go out to make sure that I advocate for the families if there are any issues that have arisen. (Cape York, Health Worker, #20*) *Yeah, [we are] like a community representative, and a vital one at that, because the (A&TSIHW) know how to explain situations, both in clinical [care] and especially if there’s an emergency - explain it at a level where [community and family] can understand so they don’t get their back up. (Cape York, Health Worker, #4*) *I’ve had nurses that have come in, I even had a doctor that has come in and forced us to do things with our in-laws or with clients - yes, really breaching big time our cultural protocol. And I’ve told them off and they don’t like me because of it, but I’ve stood up for other (A&TSIHW) as well. (Torres and NPA, Health Worker, #40*)

A&TSIHW, Aboriginal and Torres Strait Islander Health Worker; NPA, Northern Peninsula Area.

### Role orientation

While in mainstream health systems, professional roles are typically defined in terms of what they do (ie, their function), such an approach is inadequate for fully explaining the distinctive nature of the A&TSIHW role. To comprehend the role fully, we must not only understand its (often fluid) functions, but also its defining *orientation*. As represented at the centre of the triangle in figure 1, the orientation of A&TSIHW role is defined by its central concern for clients’ needs and a strengths based approach to daily practice as described further below.

#### Client centredness

No matter the functional focus of their position, participants working as A&TSIHWs in this study described the well-being of their clients as their anchoring concern. Client centredness was typically described in terms of a sense of purpose or mission, to help or ‘give back’ to their community. Highlighting its profound importance, this purpose or mission was linked by many individuals to their broader motivation for pursuing a career as an A&TSIHW.

While I was a wardsman, I saw the Health Workers and how they operate, and that got me interested in doing it. […] I’m here now as a health worker […] I like it, I am doing something for my community. (Torres & NPA, Health Worker, #33)

But I really get that passion to help people in every way, educate. That’s me. Even if I off job and if I even look someone sick, I must sit and talk. (Torres & NPA, Health Worker, #34)

I used to get up to a lot of mischief, when I was young, and the community was good to me, so I thought: “Well, it’s time for me to give back.” That’s what motivates me. (Cape York, Health Worker, #13)

Well, I should have been a nurse years ago. Yeah, yeah.[but…] all I worried about is looking after the people. (Torres & NPA, Health Worker, #35)

I loved what I did and because we were there for the people of the community, of the Cape. (Cape York, Health Worker, #27)

We're here to provide health and to me, that’s why I became a Health Worker, is to provide health. Understanding what health is. What is it that I can do in my role to provide that health? (Cape York, Health Worker, #20)

I would tell you: Listen, my role as a health worker is to help you to help my people. (Cape York, Health Worker, #1)

#### A strengths-based approach

A strengths-based approach to daily practice was how those working as A&TSIHWs operationalised this client centredness. Askew *et al*^23^ applying Martin and Mirraboopa’s^24^ framework to health service delivery, describe an Indigenous strengths-based approach as simultaneously encompassing a ‘**Way of Being**’—an everyday practice of starting with strength; a ‘**Way of Doing**’—strengths-based approaches as a relational principle; and a ‘**Way of Knowing**’—resistance against racialising practices. We used this frame to organise and illustrate the distinctive features of A&TSIHW practice that emerged from participants’ accounts.

##### Way of Being

Describing a Way of Being, many A&TSIHW participants’ narratives stressed the importance of empathy, anchored by a need to put themselves in their clients’ shoes in order to ‘feel what they’re feeling’. Strongly linked to the value placed on client centredness, A&TSIHWs described the importance of patience and being willing to take the time to build a foundational relationship with clients.

It’s just a whole ripple effect, if you get it right and you be patient and you do it right, you’ll get this whole ripple effect of success. (Cape York, Health Worker, #13)

Some participants additionally made the distinction between an approach to the client–provider relationship built on empathy versus sympathy, implying that true empathy was difficult for non-Indigenous providers who had not ‘walked in their (Aboriginal and Torres Strait Islander clients’) shoes’. The same participants explained that sympathy, while well-intended, was often received as condescending and disempowering.

A [Aboriginal and/or Torres Strait Islander] Health Worker to me is somebody helping the people, but just a little bit extra; and not sympathy but empathy. You’re feeling what they’re feeling. That’s the only way you are going to really understand. (Torres & NPA, Health Worker, #60)

Importantly, empathy and understanding were framed as not just being about the individual client, but about clients' family and community too. This Way of Being rejects the idea that healthcare is delivered to individuals alone. These views reflect some of the foundational definitions of Aboriginal and Torres Strait Islander health as articulated in the watershed 1989 National Aboriginal Health Strategy which described health as not just the physical well-being of an individual, but the social, emotional and cultural well-being of the whole community.^25^ Acknowledgement of the interconnected and interdependent nature of individuals and recognition of the need to understand and engage with a broader group of people in their lives is embedded in A&TSIHWs’ practice.

It’s understanding and empathy also for the family, not only for the patient. But you got to put yourself in their predicament. (Torres Strait & NPA, Health Worker, #61)

##### Ways of Doing

Reference to strengths-based approaches as a Way of Doing were also common in A&TSIHWs’ accounts. One participant explained how her observation of such an approach among her seniors was a key motivator to become an A&TSIHW in the first place.

I thought then: “I want to be a Health Worker”. I really want to be a Health Worker. And just seeing how these people [senior A&TSIHWs] were very strong in their roles. Seeing that the leadership and the collaboration that I saw in a cultural way, and then it made me realise where I come from, it’s totally different. […] Then I realised that I can take this wherever I go, regardless of where I am. (Cape York, Health Worker, #20)

Reflecting on a common experience of being given advice to pursue nursing careers, several A&TSIHWs also reflected how their Way of Doing, a proactive approach to improving the health of their clients, was part of what made the role different from, and more attractive than, nursing in particular:

I was told to do nursing, but I rejected that […] That’s not me, even though I do help nurses a lot. But I rather stay a [Aboriginal and Torres Strait Islander] Health Worker. I feel that I want to help them [clients] before they go to lie on the bed and get more sick. I want to help them “You must come, we’ll go for walk today.” or “Come with me, we’ll go fishing.” steer their mind off things. (Torres and NPA, Health Worker, #55)

Still others provided accounts of how they employed a strengths-based Way of Doing to advocate for their clients, even challenging dominant medical-model adopted by mainstream service providers, such as in this account:

There would be an overweight person come in, and his sugar was high. And [the doctor] would immediately class them as: “You’re a diabetic. I’ll put you on Metformin.”

And when they’d come out, and I [would always ask]: “What did the doctor say?”

Because [me and the doctor] were forever arguing. I’m like: Tell them to lose weight! Tell them: ‘Come back in three months. Like, give them a chance to change because he’s only overweight. There’s nothing else wrong with him. He has no other underlying factors.’ (Torres & NPA, Health Worker, #40)

Underpinning such an approach was the imperative to recognise and respond to the needs of the person as a whole, rather than to their ill-health or disease.

Well I just like to keep it plain and simple, like what Aunty [x] told us. Just treat the person, don’t just treat the sore, yeah. (Cape York, Health Worker, #13)

As a health worker, I feel that we can play that role too, as a big brother or you know, and just to support these young fellas. Even the elders too, because we don’t just focus it, like acute care, it’s more holistic approach. (Cape York, Health Worker, #14]

This understanding of health was described by many as being at the heart of A&TSIHWs’ distinctive, strengths-based practice. But the setting in which such practice occurred was important. Many participants described how this way of working required the space and autonomy to work in non-clinic settings. Community outreach, home visits and out-of-hours services were all examples given of Ways of Doing that centred on the needs and priorities of whole person and community.

More effective? I think it’s the home visits. […] I said […] “Boys, come on. You can't come to the clinic? I'm bringing the clinic to you.” So I sit down and do your urine and blood, then we get a swab. They enjoy that, yeah. (Cape York, Health Worker, #15)

It’s more out in the community. Like, we’ve had four deaths like one after the other. So sometimes it’s during work. Most of the time, it’s after hours where you just sit and yarn with family, like: “How’s everybody going? And everything’s good?” Like you talk to the in-laws. You talk to the people running the bereavement, and you talk to them. And then they’ll say: “Oh, can you talk to so and so?” And so you sit […] You go and you show your respects to the family. But while you’re there, you sit and yarn, and people just unload anyway. (Torres Strait & NPA, Health Worker, #37)

To say five o’clock comes: “Oh I’m sorry but I’ve got to go”. No! Torres Strait & NPA, Health Worker, #39)

##### Ways of Knowing

Strengths-based approaches as a Way of Knowing were also evident in the accounts of individuals working as A&TSIHW, and linked strongly to the earlier described motivations to serve and help their community. Askew *et al*^23^ describe a Way of Knowing as ‘*a means by which Indigenous people could assert their humanity, in order to be seen as real people, not just clients*’. The same authors go on to note that ‘*much more than a matter of a choice between good or bad stories or stereotypes, a strengths-based approach was a conscious emancipatory practice, which rejected laying blame upon Indigenous peoples for the structural conditions that impinged upon their everyday lives*’.^23^

Descriptions of precisely this type of rejection of blame were evident in accounts of several participants who articulated the need for mainstream health services to better acknowledge the (still recent) impact of colonialism and segregation on Aboriginal and Torres Strait Islander peoples’ health, and to reconstruct social and professional relationships that actively challenged structural racism.

We’re going to come out from under the thumb. Get out from under the thumb. We’re better than being under the thumb. That’s what we’re trying to do, we’re trying to help ourselves to help our people. (Cape York, Health Worker, #2)

We're never going to close the gap if we're not going to give people the ownership to take on their own health. […] I feel that our people, they can be independent if you let them. [but] because we've got a non Indigenous front, people who come on board who aren't aware of the cultural side of stuff and do not speak to the cultural person that should be advocating for the community, this becomes a barrier, and that’s part of our Closing the Gap. (Cape York, Health Worker, #20)

Pride in the continuity and strength of cultural tradition was also evident in participants’ accounts. For some this pride meant positioning cultural and familial obligations as more important than accountabilities established by their health service relationships.

We can handle the accountability and the clinical governance and all that. And that’s only taught and lost when programs go down or government changes and stuff like that - but our culture and tradition built into us will never change. Never. Even throughout my time in [a different government service], I still reacted or was obliged to community. (Torres Strait and NPA, Health Worker, #46)

For some, these ways of knowing contributed to frustration with being excluded from forums and processes that inform official policies and strategies regarding Close the Gap, observing their own knowledge and experience could and should play a role in front-line service design and implementation:

They’re not closing the gap. All we need is - you come and talk to us [about] how we can close the gap! We have ideas that we could tell you of closing the gap! (Cape York, Health Worker, #28)

Although observing the frequent lack of recognition and support for their work, participants also often reported how strength-based Ways of Knowing underpinned their near-continual effort to educate and reorient non-Indigenous professionals to better respond to the complex needs of local communities.

The stuff that I tell the doctors, the registrars [from out of town]: “You need to get cultural awareness, you need to understand.” It’s not the same [here]. You might get trained there at [university] in cultural practice awareness or whatever they do there. They give you this basic training. But when it comes to communities it’s totally different. Every community is different from what you’re told there. So when you come to my community I will tell you how you deal with this. (Cape York, Health Worker, #2)

That’s the thing with RNs when they come out, they don’t listen. Because it’s how they treat the people [like]: “I do this because I’ve got a higher degree.” And that’s what I told him. I said: “But culturally you don’t. Culturally I have a higher degree. I think your calls can be nothing. Your calls depend on the people and how they see you”. (Torres Strait and NPA, Health Worker, #38)

I’ve said to them that […] I understand [some cultural traditions] are a barrier. But we need to find a way around […] And I was told: “you need to stop this cultural shit.” So that’s when I lost my plot. How dare you come in to my community and say to us that we need to stop this. If you don’t want to be here, you can pack up and leave. (Torres Strait and NPA, Health Worker, #41)

In this way, Indigenous Ways of Knowing also served as a mechanism for challenging the lack of self-awareness, and presumption of authority by some non-Indigenous providers, reminding them that the culture of mainstream health system, rooted in Western, neoliberal and biomedical knowledge models, was neither sufficient nor always appropriate in addressing the health needs of Aboriginal and Torres Strait Islander clients.

## Discussion

*Biomedical research is free to focus on the efficacy of particular practices rather than on establishing the validity of the practitioners. Much of the literature about and by [Aboriginal and Torres Strait Islander] health workers demonstrates that deep knowledge of community informs an Indigenous practice and cannot be simplistically tethered to a set of biomedical ‘competencies*’. Bond *et al*, 2019^26^

*…health is not dependent on the physical wellbeing of individuals […] When considering health, you need a model that has a focus on structural inequalities, not just a focus on personal stories of misfortune. Also you need a model that acknowledges a history of oppression and dispossession, and a history of systemic racism*. O’Donoghue, 2007^27^

Although not always explicit in state-based policies and frameworks, the A&TSIHW role is one of only two ‘identified’ positions in the mainstream Queensland health workforce and the only role with explicit responsibility for advocating culturally safe care at the facility or service level. Despite the significance of these responsibilities, evidence from the peer-reviewed literature,^28–30^ and the broader project of which this study forms a part,^31^ demonstrates pervasive lack of recognition of the A&TSIHW role in industrial, organisational and professional domains. Poor recognition of A&TSIHWs stems from a health service structure that privileges certain knowledge and roles, and which enables non-Indigenous policymakers, managers and broader health workforce to avoid engaging with the true purpose, function and orientation of the role should they wish. Consciously positioned to represent the perspective of those working as A&TSIHW, this paper reports on the role’s core functions and distinctive orientation, and seeks to articulate once again its value at the intersection of clinical service delivery, health promotion and brokerage in 21st century Australian healthcare.

Participants’ accounts highlight the multifaceted nature of the A&TSIHW role, which demands simultaneous activity (although with various emphasis) across the three core functions of health promotion, clinical service and cultural brokerage. In the context of what most A&TSIHW participants in this study saw as their professional purpose (ie, to improve clients’ health through a person-centred focus) the fluid and multifaceted functions of their role made sense. Participants explained how the cross-cutting functions helped them place the person—rather than disease—at the centre of their work, reminding them to give attention to the cultural, social and environmental contexts in which their clients’ health was constituted. These cross-cutting functions aligned with the role-specific qualifications and formal role description both which emphasise application of a primary healthcare approach^4^ and hint at (if not fully enabling) a model of care that accounts for structural inequalities rather than just ‘personal stories of misfortune’.^27^ Articulating great pride in their profession and these distinctive skills, A&TSIHWs in this study stressed that they did not view their role as ‘lesser’ than other health professional roles (particularly nursing) but as distinct and complementary to such professions. However, embedded in a health system that rewards medical specialisation and conducts workforce planning predicated on splicing and grouping professionals to create ‘optimal skills mix’,^32 33^ the fluid, multifunctional (generalist) nature of the A&TSIHW role is increasingly an anomaly.

As powerfully articulated in the quote by Bond *et al*^26^ at the beginning of the Discussion ‘*deep knowledge of community informs an Indigenous practice and cannot be simplistically tethered to a set of biomedical ‘competencies*’’. Underpinning the cross-cutting functions of the A&TSIHW role, participants emphasised its unique orientation, defined by client centredness and realised through Indigenous strengths-based ways of knowing, being and doing. These findings have resonance with those reported by Dickson^18^ whose rich descriptions of the experiences of 15 A&TSIHWs highlight the seamlessness of working and living in the same community as an important feature of their work and vital to providing quality health services to their community. Both Dickson and the current study highlight how at the core of differences between A&TSIHW and other health professional roles, lies an ability to, and responsibility for, holding different knowledges and considering the contexts and manner in which each should be invoked to provide high-quality physical, mental and spiritual care for clients. Uniquely within the Australian setting, the A&TSIHW role is thus a mechanism through which active adaptations are being made to otherwise biomedically-driven work practices in order to account for the different knowledge and value systems that shape understandings of health of Aboriginal and Torres Strait Islander peoples.

Recognition of the need for this type of improved cultural responsivity within the dominant biomedical model of Australia’s health service is evident in publication of national and state-level policies, frameworks and strategies targeting improved cultural awareness.^5 34–36^ Yet of the Australian context, Baum *et al*^37^ note that ‘*…medical perspectives prove the most powerful and are reinforced by the actors, ideas and institutions that shape [primary health care]. Community perspectives which stress lived experience and social perspectives on health are marginal concerns*’. Of Aboriginal and Torres Strait Islander health more specifically, Durey and Thompson observe that healthcare and health systems in Australia are ‘*racialized social structure[s] where Indigenous knowledge, beliefs and values are subjugated to the dominant western biomedical model in policy and practice*’.^38^

These observations underpin a distinction made by Aboriginal and Torres Strait Islander scholars between ‘cultural competence’ or ‘awareness’ on the one hand, and being ‘culturally *responsive*’ and promoting cultural safety on the other.^39–42^ As synthesised by Flemington and Fraser^43^ cultural competence locates power in the professionals’ hands to determine what competent care is, and predicates service delivery on ‘treating everyone the same’. Being culturally responsive and promoting service-wide cultural safety on the other hand requires health professionals to actively respond to cultural differences^43^ and engage in critical thinking and self-reflection in order build understanding of, among other things, the way (dominant) cultures and value systems inform practice that may negatively impact the quality and acceptability of services.

The dominance of medical knowledge and medical professionals, and marginality of Aboriginal and Torres Strait Islander knowledge systems and their carriers, is the critical institutional context in which A&TSIHWs employed in government health services operate and survive.^44–46^ A&TSIHW in this study described a daily practice grounded in the need to continually navigate and mediate cultural tensions within a health system that normalises racism and assimilation, and defines biomedical knowledge as superior. Yet through their strengths-based practices, many of which challenge assumptions about what is acceptable and appropriate healthcare in a local context, A&TSIHWs simultaneously model cultural responsiveness and encourage non-Indigenous colleagues to engage in critical self-reflection.

Building on previous work,^18 26 47 48^ findings in this study thus strengthen the evidence base regarding the unique contribution of A&TSIHW to Australia’s mainstream health workforce. At least in the rural and remote setting in which this work was carried out, the A&TSIHW role is the only professional role with both a corporate remit *and* the cultural expertise to execute the intent of national and state-based cultural safety policies by modelling and promoting greater cultural responsiveness within front-line services consistently and on a day-to-day basis. As reports of institutional racism within Australia’s government health services continue to surface,^45 49 50^ the importance of such a role is critical.

But acknowledgement of the personal and professional cost to individuals of carrying out this work is also important. Among A&TSIHW participants in this study, accounts contained descriptions of near-daily conflict, resulting from the need to identify, confront and mitigate tensions between non-Indigenous service providers and Aboriginal and Torres Strait Islander clients. Notwithstanding significant research and theorisation of work done at the cultural interface^18 51^ structural recognition of, or respect, for the complexity (and trauma) of such roles remains sadly lacking, and exemplified in the continued lack of resources for dedicated interpreter services^50^ in many Aboriginal and Torres Strait Islander language-speaking regions of northern Australia.^52^ Such experiences, as will be described in more detail in future manuscripts, contribute to burn out and high turnover within the A&TSIHW profession, particularly in remote communities.^21 53 54^

## Conclusion

Our findings highlight the multifaceted nature of the modern A&TSIHW role, which demands simultaneous activity (although with various emphasis) across the three core functions of health promotion, clinical service and cultural brokerage. Underpinning the cross-cutting functions of the A&TSIHW role, participants emphasised its unique orientation, defined by client centredness and realised through Indigenous strengths-based ways of knowing, being and doing. Findings highlight the unique nature of the A&TSIHW role as a mechanism through which Aboriginal and Torres Strait Islander knowledge can be brought to bear on context-specific service adaptations in the mainstream government health service; and through which the impacts of lack of cultural or self-awareness among some non-Indigenous health professionals are being mitigated. Urgent consideration should be given to the way poor structural recognition and respect for the complexity of this work, within the health system where a dominant biomedical culture defines what is valued and therefore resourced, is potentially threatening the long-term viability of the A&TSIHW role.

## Data Availability

No data are available. Data were collected under strict conditions of confidentiality and protection following human research ethics guidance for Aboriginal and Torres Strait Islander research provided by National Medical Research Council.
